# Quality of life of patients after retropubic prostatectomy - Pre- and postoperative scores of the EORTC QLQ-C30 and QLQ-PR25

**DOI:** 10.1186/1477-7525-9-93

**Published:** 2011-11-02

**Authors:** Peter Bach, Tanja Döring, Andreas Gesenberg, Cornelia Möhring, Mark Goepel

**Affiliations:** 1 Department of Urology of Klinikum Niederberg Velbert, Academic Hospital, University of Duisburg-Essen, Germany

## Abstract

**Background:**

Patients with newly diagnosed early stage prostate cancer (PCa) face a difficult choice of different treatment options with curative intention. They must consider both goals of optimising quantity and quality of life. The quality of life (QoL) is a psychometric outcome which is measured using validated questionnaires. Only few data are published concerning pre - and postoperative QoL.

**Methods:**

This study investigated pre perative QoL of 185 patients who consecutively underwent open radical retropubic prostatectomy for organ-confined PCa to postoperative QoL of another 185 patients. The EORTC QLQ-C30, EORTC QLQPR25 module and 24 h ICS pad test were used (mean follow-up 28.6 months).

**Results:**

The examined symptom scores of the EORTC QLQ-PR25 were on lowest level. In the dyspnoea symptom score differences of age emerged: the amount of patients who are short of breath rose significantly in older patients after surgery (p < 0.05 paired, two-tailed student's t-test).. Lastly, the urinary symptom score was found postal-therapeutically low; this fact was age independent. The results of sexual symptom score need to be taken into consideration, since prostatectomy resulted in a significant reduction of sexual activity independent of age. All functioning scales postoperatively reached high values without significant changes (p > 0.05 student's t-test ), which implies a high QoL after surgery. A reliable and satisfying status of continence was found in our patients after retropubic prostatectomy. A high rate of patients (89.2%) would choose retropubic prostatectomy again.

**Conclusion:**

Retropubic prostatectomy represents a reliable and accepted procedure in the treatment of organ-confined PCa. For the first time it could be shown that patients` QoL remained on a high level after retropubic prostatectomy. Nevertheless, the primary avoidance or postoperative therapy of erectile dysfunction should be in the focus of surgeons.

## Background

Prostate cancer (PCa) is the most common malignancy in men worldwide and is actually detected as localized disease in most patients. Diagnosis and therapy of PCa has long-ranging consequences for patient's further life. Patients with newly diagnosed early stage prostate cancer face a difficult choice of different treatment options with curative intention, and they must consider both goals of optimising quantity and quality of life. Radical prostatectomy is regarded as a standard surgical treatment in organ-confined PCa and may be performed in a retropubic, perineal, laparoscopic or robotic-assisted way. Radiotherapy is established as a non-surgical approach in the curative treatment of localized PCa in its variations of external beam radiation, brachytherapy and permanent seed implantation. Recently research efforts have been made to sharply focus on showing and measuring quality of life outcomes together with more traditional end points of survival and disease-free status [1-4].

Despite advances in surgical techniques, the most common adverse consequences of radical prostatectomy continue to be urinary incontinence, erectile dysfunction and anastomic stricture [3,5]. QoL is a psychometric outcome which is measured using validated questionnaires. In our study the EORTC QLQ-C30 and EORTC QLQ- PR25 module [6] were used.

## 2. Methods

The main aim of this study was to compare QoL before and after radical prostatectomy. Until today it remains unclear whether radical prostatectomy leads to measurable postoperative morbidity and therefore influences QoL. Validated pretherapeutical QoL scores are still missing. However, this data is needed to help patients choosing between different therapeutic surgical and also non-surgical options. In addition, additional and different surgical approaches have been developed during the last years (laparoscopic and robotic-assisted laparoscopic surgery), which seem to lead to comparative oncological results with possible lower change of QoL. Thus QoL may be a major factor in comparing different surgical procedures [3].

### 2.1 Study design: Prospective clinical trial

On the day before radical retropubic prostatectomy the EORTC QLQ-C30 and EORTC QLQ-PR25 module was filled out by patients (n = 185) for preoperative QoL data (2006 to 2008). A matched-pairs analysis was performed regarding age. At least 6 months after surgery another 185 patients (who were matched-pairs regarding age) were subjected to the EORTC QLQ-C30 and EORTC QLQ-PR25 module for postoperative QoL scores and underwent a 24 h ICS pad test (mean follow-up 28.6 months) (2002 to 2006). During hospital stay after surgery and catheter removal a 24 h ICS pad test had been performed before [6-8]. For the match-pairs analysis no disease characteristics or nerve-sparing procedures were used. Ethical approval was asked at University of Essen during 2005.

### 2.2 Patients

This study investigated two groups of 185 patients each who consecutively underwent open radical retropubic prostatectomy at the Department of Urology of Klinikum Niederberg, Velbert, Germany. Three surgeons performed ascending retropubic prostatectomy including regional lymphadenectomy of the regions arteria iliaca externa, interna, and obturatoric area.

Average patient age was 66.5 years (range 48 to 79 y). 134 patients (72.4%) were younger than 70 years, and 51 (27.6%) older than 70 years at the time of surgery. In guidelines of german association of urology (DGU) in 2004 there has been a restricted recommendation for prostatectomy in patients 70 years or older. Therefore all datas were analyzed in subgroups younger and older than 70 years. Since 2006 the QLQ-C30 plus QLQ-PR25 module was assessed for our patients before prostatectomy.

In order to perform a comparison between pre- and postoperative QoL data, a matched-pairs analysis regarding age was performed between preoperative data from 185 patients who underwent surgery from June 2006 to June 2008 to postoperative data from 185 patients from 2002 to 2006.

The amount of nerve-sparing prostatectomy was different in both groups (32,4% (60/185) to 36,7% (65/185)).

### 2.3 Quality of life

#### 2.3.1 Assessment of quality of life

Prostate cancer-specific EORTC QLQ-PR25 module is available since mid-2006. We carried out a matched-pairs analysis in our presurgical population. A matched-pairs analysis is a statistical procedure assigning a control person to every patient. In this study the matched patient was chosen with the same age on the day before radical prostatectomy.

EORTC QLQ-PR25 questionnaire was performed on day before surgery and during follow-up at a minimum of 6 months after surgery (mean 28.6 months).

#### 2.3.2 EORTC QLQ-C30

EORTC QLQ-C30 is a validated questionnaire for QoL in patients suffering from malignancy [8,9]. EORTC QLQ-C30 measures QoL and general status of health in a score called Global Health Status (GHS), allowing values in a range from 0 to 100. Therefore, high scores represent a high QoL and low scores a low one. Five functional scales measure body, role, emotional, cognitive and social function of patients. Again, a high value reflects high function of functional scales and low value shows low or disappointing function.

Additionally, the questionnaire includes three symptom scores (fatigue, emesis and pain) and six further single-item symptom scores (dyspnoea, insomnia, appetite loss, constipation, diarrhoea and financial difficulties) which may occur in PCa patients. All these scales and scores have four scoring possibilities, ranging from 1 (not at all) to 4 (very often). A high symptom score represents a large amount of symptoms. For better classification all scores and items are shown on scales from 0 to 100 [7].

In addition to the EORTC QLQ-C30 as a basic questionnaire few additional modules are published, which access different malignancies or states of disease. We used the PR 25 module which is avaibale since 2006. It was published in 2008 as a validated tool for PCa [6].

#### 2.3.3 EORTC QLQ-PR25 Module

An additional questionnaire contains four symptom scales which are of main interest in evaluating QoL. These are symptoms concerning defacation, micturition, treatment and sexuality (bowel-, urinary-, treatment-related and sexual symptoms) [6].

Postoperative urinary symptom score is of main interest to surgeons because of its important role in patients' QoL. It is important to know that questions scoring for urinary symptoms are valuing urge incontinence more than stress incontinence. Stress incontinence after radical prostatectomy was evaluated by ICS 24 h pad test (see 2.4). Sexual symptom scores were not comparable, because of a different amount of a nerve-sparing prostatectomy in both groups (data not shown).

### 2.4 International Continence Society 24 hours pad test

Patients were instructed to weigh dry pads, collect wet pads and weigh wet pads after 24 h. This test was performed under daily life conditions [10].

ICS 24 h pad test was performed within 12.2 days after surgery. Late continence was assessed by another 24 h pad test 6 or more months after surgery. Patients reporting pad usage were followed up to 28.6 months.

### 2.5 Self-created questionnaire

Patients were asked another four questions in addition to validated questionnaires which could be answered by "Yes" or "No". These answers were used for assessing the quality of treatment and the degree of patients' satisfaction concerning treatment (median follow-up 28.6 months). For further details see Additional file 1.

### 2.6 Statistics

Microsoft Excel 2002™ was used for surveying data and performing matched pairs analyses. Significance was calculated by parametric and paired t-tests (Wilcoxon signed rank test and paired ANOVA followed by Bonferroni's multiple comparison test) using GraphPad Prism™. A p < 0.05 was regarded as significant.

## 3. Results

### 3.1 Quality of Life

#### 3.1.1 Global Health Status

The patients' state of health and quality of life assessed by self-evaluation is regarded as the *global health status *(GHS). Patients older than 70 years showed acceptable value for GHS following surgery of 69.3 with a significant reduction to preoperative GHS (73.5; p < 0.05, Student's t-test). A significant reduction of GHS concerning all patients was not found (p > 0.05, Student's t-test). For further details see table 1.

**Table 1:** Global health status

global health status		Patients	
	all	≤ 70 years	> 70 years
preoperative	73.8 ± 22.6	73.8 ± 22.5	73.5 ± 25.2
postoperative	69.4 ± 17.1	69.7 ± 16.0	69.3 ± 19.8*

Preoperative and postoperative global health status is shown as mean ± standard error of the mean of all patients and patients' ≤ 70 years and > 70 years (matched-pairs analysis). * p < 0.05 vs. preoperative value, paired, two-tailed student's t-test

##### 3.1.1.1 Preoperative global health status

A total of 104/185 patients (56.8%) showed state of health as good to excellent one day before surgery (56.9% > 70 years vs. 56.0% ≤ 70 years). A bad to poor state of health was described by three patients (1.6%). 48.1% patients showed QoL good to excellent (48.1% > 70 years vs. 48.5% ≤ 70 years; p > 0.05, Student's t-test), 6% reported bad to poor quality of life before surgery (5.9% > 70 years vs. 6% ≤ 70 years; p > 0.05). No significant difference in preoperative global health status and quality of life was seen between age groups as assessed by student`s t-test (p > 0.05).

##### 3.1.1.2 Postoperative global health status

58.4% (108/185) patients showed state of health as good to excellent 6 months after surgery (59.7% > 70 years vs. 55.0% ≤ 70 years; p > 0.05 student's t-test). 32.5% of patients reported good to excellent quality of life postoperatively (23.5% > 70 years vs. 35.9% ≤ 70 years; p > 0.05 student's t-test).

Regardless of age six patients (3.2%) reported bad to poor quality of life at least six months following surgery (p > 0.05 student's t-test).

#### 3.1.2 Functioning scales of EORTC QLQ-PR25

Cognitive and social functioning scales pointed a high level of functioning (> 90) before surgery with significant changes (all p < 0.05 Student's t-test) following prostatectomy. Emotional functioning scale showed a low level one day before prostatectomy (78.2) and a significant higher score (90.4) during follow-up independent of age (all p < 0.05 Student's t-test).

Preoperative sexual functioning scale represented the lowest function level (55.7). There was no significant difference after treatment (p > 0.05 student's t-test). For further details see table 2.

**Table 2:** Functioning scales of EORTC QLQ C30

functioning scales	patients		
	all	≤ 70 years	> 70 years
pre physical functioning	93.2 ± 9.9	93.5 ± 9.1	92.4 ± 11.7
post physical functioning	94.4 ± 11.6	94.7 ± 10.0	93.5 ± 15.0
pre role functioning	92.2 ± 13.4	92.9 ± 12.8	90.5 ± 14.5
post role functioning	91.4 ± 18.6	92.5 ± 16.9	88.9 ± 22.1
pre emotional functioning	78.2 ± 22.7	76.4 ± 24.4	80.9 ± 18.9
post emotional functioning	91.4* ± 14.3	91.2* ± 15.1	90.4* ± 15.2
pre cognitive functioning	91.6 ± 16.7	92.2 ± 15.5	90.5 ± 17.2
post cognitive functioning	94.6 ± 11.3	95.2 ± 9.5	93.5 ± 14.8
pre social functioning	90.2 ± 14.4	90.2 ± 14.4	89.9 ± 14.4
post social functioning	91.5 ± 19.4	91.2 ± 19.9	90.5 ± 20.7
pre sexual functioning	55.7 ± 32.3	54.3 ± 31.7	59.0 ± 33.2
post sexual functioning	56.8 ± 30.0	55.5 ± 30.5	59.6 ± 28.5

Pre (pre) - and postoperative (post) data are shown (mean ± standard error of the mean) for physical, emotional, role, cognitive, social and sexual functioning. n = 185 for all scales paired, two-tailed student's t-test * p < 0.05 vs. preoperative values

#### 3.1.3 Symptom scores of EORTC QLQ-PR25

Patients pre- and postoperatively scored low values represented few or a total lack of symptoms. Only the score for sexual symptoms showed higher values. For further details see Table 3.

**Table 3:** EORTC QLQ-PR25 Symptom scores

symptom scores	all patients	patients ≤ 70 years	patients > 70 years
preoperative fatigue	7.5 ± 12.6	7.9 ± 12.8	6.1 ± 11.7
postoperative Fatigue	6.4 ± 13.7	4.8 ± 10.3	10.2 ± 19.4
preoperative nausea & vomiting	1.00 ± 4.0	0.9 ± 3.7	1.3 ± 4.5
postoperative nausea & vomiting	1.00 ± 5.8	0.6 ± 4.3	2.0 ± 8.5
preoperative pain	8.3 ± 16.2	6.8 ± 13.7	11.8 ± 21.0
postoperative pain	9.0 ± 16.7	7.6 ± 14.6	12.4 ± 20.6
preoperative dyspnoea	7.8 ± 16.5	7.2 ± 15.4	9.2 ± 18.8
postoperative dyspnoea	15.3* ± 23.0	13.7 ± 20.9	19.0* ± 27.4
preoperative insomnia	7.8 ± 15.4	7.7 ± 15.2	7.8 ± 15.6
postoperative insomnia	15.5* ± 25.6	16.7* ± 26.0	11.8 ± 23.6
preoperative appetite loss	4.6 ± 11.5	5.5 ± 12.4	2.0 ± 7.8
postoperative appetite loss	3.1 ± 15.1	2.5 ± 13.9	4.6 ± 17.5
preoperative constipation	5.8 ± 12.7	6.0 ± 12.8	5.2 ± 12.1
postoperative constipation	6.4 ± 18.2	6.2 ± 17.4	6.5 ± 19.8
preoperative diarrhoea	2.9 ± 9.4	2.7 ± 9.2	3.3 ± 9.9
postoperative diarrhoea	2.7 ± 11.5	2.0 ± 10.6	4.6 ± 13.2
preoperative financial difficulties	0.6 ± 4.2	0.8 ± 4.9	0.7 ± 4.6
postoperative financial difficulties	1.6 ± 8.7	1.2 ± 7.5	3.3 ± 11.9
preoperative urinary symptoms	14.1 ± 15.1	14.1 ± 15.5	13.7 ± 15.0
post urinary symptoms	9.1 ± 11.8	9.8 ± 12.2	7.1 ± 10.4
preoperative bowel symptoms	1.1 ± 3.9	1.0 ± 3.2	1.5 ± 5.1
postoperative bowel Symptoms	2.3 ± 7.3	1.9 ± 6.4	3.1 ± 9.0
preoperative treatment-related symptoms	8.1 ± 8.8	8.7 ± 9.0	6.2 ± 7.5
postoperative treatment-related symptoms	11.5 ± 10.6	12.0 ± 10.8	10.1 ± 9.5
preoperative sexual symptoms**	32.2 ± 30.8	32.6 ± 32.1	29.2 ± 26.3
postoperative sexual symptoms***	45.3 ± 20.4	48.3 ± 19.7	35.2 ± 19.7

Pre - and postoperative values of all sympotm scores (mean ± standard error of the mean; n = 185) as a match-pairs analysis, paired, two-tailed student's t-test

* p < 0.05 vs. preoperative values

** n = 127 patients

*** n = 52 patients

##### 3.1.3.1 Dyspnoea symptom score

The dyspnoea symptom score rised from a low level preoperative to higher postoperative levels for the whole study population (from 7.8 ± 16.5 (mean ± s.e.m.) to 15.3 ± 23; p < 0.05 paired, two-tailed Student's t-test). Patients > 70 years suffered from significant higher scores in postoperative analysis (p < 0.05 student's t-test).

##### 3.1.3.2 Insomnia symptom score

Insomnia symptom score changed significantly from preoperative to postoperative population (7.8 ± 15.4 (M ± SEM) to 15.5 ± 25.6; p < 0.05 student's t-test).

##### 3.1.3.3 Urinary Symptom Score

Pre - and postoperative urinary symptom scores of all patients showed no significant difference (p > 0.05 student's t-test). A subgroup analysis of patients suffering from a high-grade incontinence (II° and higher; n = 11) showed an average GHS (70.3) (p > 0,05; student`s t-test following Bonferroni's multiple comparison test).

##### 3.1.3.4 Sexual Symptom Score

Because of the different amount of patients underwent a procedure of nerve-sparing prostatectomy in both groups a valuable comparison of pre - and postoperative sexual symptom scores could not be performed.

A subgroup analysis found in the sexual active patients (52/185) a high QoL (73.4). In comparison to the whole postoperative population we found a significant difference (p < 0.08; students t-test). 78.8% (41/52) of the postoperative sexual active patients received a nerve-sparing procedure.

### 3.2 Continence

The status of continence resulting from ICS 24 h pad test was processed as a multivariate analysis to life age, blood loss and TNM stadium. A predictive factor for incontinence following prostatectomy could not be found. Due to the relatively low number of patients, a valid analysis of continence concerning histological classification could not be performed.

#### 3.2.1 Status of early continence

Early ICS 24 h pad test reported 69.2% (n = 128) of all patients as primary continent. The number was not significantly different with respect to patient's age (older than 70 y: 70.6% vs. younger than 70 y 60.7%; p > 0.5, student's t-test following Bonferroni's multiple comparison test).

In 16 patients with high grade incontinence (II°-III°) no significant difference was found concerning patient's age (older than 70 y: 7.8% vs. younger than 70 y 8.9%; p > 0.5 student's t-test following Bonferroni's multiple comparison test). For further details see table 4.

**Table 4:** Results of early continence

	continence		I°-II°	II°	II°-III°	III°
Pads/24 h	No	Safety pad	1-2	2-4	5-8	> 8
ICS Pad Test (ml)	0	0-2	2-10	10-50	> 50	no micturition
All patients (%)	18.9	50.3	15.1	7.0	4.3	4.3
Patients ≤ 70 years (%)	20.2	48.5	14.2	8.2	3.7	5.2
Patients > 70 years (%)	15.7	54.9	17.7	3.9	5.9	1.9

Results of the early continence in ICS 24 h pad test following radical retropubic prostatectomy (follow-up 12.2 days). A loss of 0-2 ml urine was regarded as social continent (n = 185; p > 0.5 between young and old patients, student's t-test).

#### 3.2.2 Status of late continence

163 patients (88.1%) reported to use no pad or only a safety pad during follow-up (28.6 months); this outcome was independent of age (older than 70 y: 80.4% vs. younger than 70 y: 91.0%; p > 0.5 student's t-test following Bonferroni's multiple comparison test). For further details see table 5.

**Table 5:** Results of late continence

	continence		I°-II°	II°	II°-III°	III°
	no pads	a safety pad	1-2	2-4	5-8	> 8
ICS pad test (ml)	0	0-2	2-10	10-50	> 50	no micturition
All patients (%)	81.6	6.5	5.9	4.4	1.1	0.5
Patients ≤ 70 years (%)	85.1	5.9	5.2	2.2	0.8	0.8
Patients > 70 years (%)	72.6	7.8	7.8	9.8	2.0	0.0

Results of the late ICS 24 h pad test following open retropubic radical prostatectomy (follow-up 28.6 months). A loss of 0-2 ml urine was regarded as social continent (n = 185). Incontinence > II° showed a significant difference and was dependent of age (p < 0.02 student's t-test).

### 3.3 Surgical results

Our study was performed in a typical population undergoing radical prostatectomy. For further details see table 6.

**Table 6:** Surgical results

Item	Median	Range
Time of operation	116 min	50 to 360 min
Follow-up	28.6 months	6 to 62 months
PSA level	9.1 ng/ml	0.3 to 59.0 ng/ml
Gleason Sum	6.3	4 to 9
No. of lymph nodes	11	4 to 24
40-day survival	100%	-
Hospital stay	13.2 days	7 to 21 days
	**Positive margins R1**	
Total		69/185 (30.9%)
pT2		15 (10.6%)
pT3		45 (64.3%)
pT4		9 (100%)

### 3.4 Satisfaction questionnaire

Nearly all patients (89.2%) would choose the surgical approach again when asked 6 months after retropubic prostatectomy. A similar large proportion of patients felt well informed about prostate cancer (86.5%). The cosmetic outcome was regarded as satisfying by about 88.1% of all patients. For these three questions no significant difference was found between age groups (p > 0.05 student's t.-test). For further details see table 7.

**Table 7:** Satisfaction questionnaire

Satisfaction questionnaire		All patients	Patients ≤ 70 years	Patients > 70 Jahre
1. Would you choose prostatectomy again?	Yes	89.2%	90.3%	86.3%
	No	10.8%	9.7%	13.7%
2. Do you feel well informed about prostate cancer?	Yes	86.5%	85.8%	88.2%
	No	13.5%	14.2%	11.8%
3. Did you receive a therapy of erectile dysfunction?	Yes	24.9%	29.1%	13.7%*
	No	75.1%	70.9%	86.3%
4. Are you satisfied by cosmetical outcome?	Yes	88.1%	88.1%	88.2%
	No	11.9%	11.9%	11.8%

Answers of 185 patients following prostatectomy during follow-up are shown. There are no significant differences between age groups (p > 0.05, student's-test) with the exception of erectile dysfunction therapy (*p < 0.05 vs. younger patients).

The number of patients who underwent therapy of erectile dysfunction was small (24.9%). In older patients, even a smaller amount of patients received treatment (13.7%, p < 0.05 vs. younger patients, two-tailed student's t-test).

## 4. Discussion

Therapy decisions may lead to cancer treatment success, but may also be followed by typical complications. Patients' satisfaction is influenced by postoperative QoL as well as by postoperative morbidity. Critical evaluation of treatment pathways is essential to reach new clarifications and better therapy decisions for patients and therapeutic options in the near future.

Recent publications regarding localized prostate cancer published by radiotherapeutics show a careful and precise assessment of QoL [5,11-13].

The first studies were published assessing QoL using the EORTC QLQ-C30 including the prostate specific QLQ-PR25 module in 2008. The PR25 module was validated in October 2008 by Aaronson and colleagues [6]. Only few studies contain data from PR25, and here data concerning open operative therapy and preoperative status are still missing [14,15].

Therefore investigation of QoL in postoperative patients is most important, because prospective randomized trials comparing different therapy pathways (e.g., operation vs. radiotherapy) are still missing. Our study investigates a patient population before and after radical retropubic prostatectomy. This data is comparable to published populations respective to age and state of localized disease [16].

Interpretation of this data in a scientific context still causes difficultly because to date only a few published studies are available with data from the EORTC QLQ-PR25. Quality of life within a retrospective analysis may rise with the number of included patients, because patients with good postoperative results more often take part in questionnaires and therefore positively influence the results. Moreover, patients with worst outcome may have died within the time of follow-up and hence unavailable to answer a survey as well. In our study records of 83% of all included patients were analyzed, which is comparable to similar studies [17].

### 4.1 Quality of life

A possible decrease of Quality of life (QoL) after RRP patients was of growing interest in recent retrospective analyses [14]. Post-therapeutic morbidity and changes of QoL are important to regard efficient cost/use analysis of cancer therapy pathways. Pre- and post-surgical state of QoL in our patients contributes therefore to the quality assurance of surgery in our department. Therefore it was focused on QoL before and after surgery.

#### 4.1.1 Global Health Status

The global health status (GHS) is a point value out of the self-assessment of the QoL of a patient. The values of GHS of patients suffering from prostate cancer in our study population are in line with published data worldwide [11,14]. A GHS of 76.3 points is described by Arredondo in 2006 that changed to 74.1 points on average two years after radical retropubic prostatectomy. In our population, the median GHS started on 73.8 points and ended up at 69.4 points. It is noteworthy that Arredondo reported about a larger (854 patients) and, on average, younger population [18], and younger patients may subjectively experience a greater decrease in QoL because of their greater overall wellbeing.

The decrease of QoL after surgery was significant only in patients older than 70 years. Here the results differ from data of Arredondo, which showed no significant change in QoL in different age groups. But, as mentioned, his population was younger on average at the time of surgery, and our patients older than 70 were twice as frequent compared to Arredondos study (27% vs. 13%). Radical prostatectomy should be discussed carefully with patients older than 70, mentioning the possibility of greater-than-average QoL loss.

However, different conclusions concerning age and QoL were drawn in history: For example. Jayadevappa showed that age of patients has no influence on QoL following prostatectomy [19]: 115 patients older than 65 years underwent either a radical prostatectomy or radiotherapy. After 3, 6 and 12 months, no reduction of the QoL could be found. Authors concluded age not determining the choice of treatment in prostate cancer. Our data show no significant reduction of Qol by a radical prostatectomy in our study population as well. Published data of GHS are on similar level to GHS scores of our patients [11].

#### 4.1.2 Functioning scales of EORTC QLQ-C30

RRP did not affect functioning scales of the EORTC QLQ-C30. There was no significant change between pre- and postal-surgical values and between younger and older patients. The only exception occurred in the emotional functioning scale. Preoperative concerns were reported by all patients independent from age. After surgery this scale significantly improved by about twelve points (78.2 to 91.4). Emotional functioning scales in published studies shows similar data [3]. Lips published comparable data concerning quality of life after radiotherapy. A significant rise of the emotional functioning scale six months after radiotherapy was seen there, which is in the same range observed by us. Successful coping strategies and temporal distance to the diagnosis may be responsible for restoration of emotional functioning. No significant change of other functional scales was observed by Lips 6 months after therapy. Also sexual functioning scale did not change after therapy independent from surgical or radiotherapy [3,11].

#### 4.1.3 Symptom scores of EORTC QLQ-PR25

Most symptom scores did not change after surgery. Only dyspnoea, insomnia, urinary and sexual symptom score showed significant changes, which will be discussed below. The data of Lips, Arredondo and Jayadevappa show similar results after therapy. Only bowel symptom score remained unchanged and increased significantly 6 months after radiotherapy [11,18,19].

##### 4.1.3.1 Dyspnoea symptom score

In our data dyspnoea symptom score increased significantly from 7.8 to 15.3 points after surgery. In the older population this change was more predominant (9.2 to 19.0 pts). Each of our patients received an preoperative chest x-ray and none of these patients suffered postoperative from an pulmonary embolism. But no further investigation were performed. Howeverwe found here a significant difference compared to published data. Lips could not detect any change in dyspnoe three years after therapy in a comparable study group concerning age and comorbidity [11]. Surgery could be responsible for this effect, because only small but significant changes (10 points) were noted. Increase of dyspnoea will influence QoL of cancer patients [20]. Patients suffering from pulmonal comorbidity need to be carefully informed.

##### 4.1.3.2 Insomnia symptom score

Compared to published data our study showed lower symptom scores concerning insomnia. In younger patients figures significantly increased after therapy.

##### 4.1.3.3 Urinary Symptom Score

32% of preoperative patients had medical treatment for bladder outlet obstruction. Questionaire dominates urge incontinence more than stress incontinence symptoms. Urinary symptom score after therapy decreased (9.1) below preoperative level (14.1). Even a higher incontinence resulting in higher urinary symptom score showed no significant reduction of QoL in our patients. Urinary symptom scores were similar to recent published data after radiotherapy (15 to 17, Lips 2008). Because PR25 could be converted only in 2008 into a phase IV module there is still a lack of validated data. Sacco et al. observed reduction of QoL by incontinence symptoms compared to age (a larger population with comparable age distribution was examined) [11,21].

QoL was not limited by bladder symptoms in our patients' independent of age and of incontinence.

##### 4.1.3.4 Sexual Symptom Score

Sexual disability caused by non nerve-sparing prostatectomy leading to a reduction of QoL is known from many investigations [4]. High sexual symptom scores (45.3) were found in our post-surgical population. Here we found the highest values of all symptom scores in our investigation. The also preoperative high sexual symptom score increased after surgery. However, this was not statistically significant. But because of an amount of 36,7% nerve-sparing procedures in this population we could not draw a conclusion in general.

The amount of patients who gained sexual activity after a nerve-sparing prostatectomy is significant higher, therefore leading to a higher QoL in this subroup.

Comparing to patients after radiotherapy referring PR25 (Junius 2007; n = 38) a similar sexual symptom score (44) was noted. A possible explanation is anti-androgen medical therapy together with radiotherapy. Conclusively, significant reduction of the sexual symptom score was seen six months after therapy (to 17.2) [22]. Lips also saw a reduction of the sexual activity after radiotherapy and a significant rise of sexual symptom scores [11].

### 4.2 Results of continence

#### 4.2.1 Status of early and late continence

69.2% of all operated patients reached continence after 12 days after surgery, and 88.1% after about 28.6 months. These results are comparable with published data of large studies concerning continence after radical prostatectomy [21].

The short-term result of continence following open surgery (6 weeks after operation) is reported to be 18-48% [23,24].

Published long time results of continence vary from 38% to 92% [16,21,25] Compared to these data, the status of early continence in our study seemed to be better and the status of late continence within average. One reason for this variance among others is a missing of a uniform definition of continence in different publications. Hence, a comparison of continence results is difficult and modestly reliable at best. Moreover, in the large studies cited here, the status of continence was mostly asked for and not raised objectively.

For example, the working group of McCammon determined status of continence of 199 patients after radical prostatectomy after 12 months. Post-prostatectomy incontinence in this study was defined by more than two self-reported incontinence episodes in 24 hours; however, a validation did not occur. 76.3% of the operated patients reported no pads, but only 38.2% indicated not to suffer from incontinence [25].

Evaluation of continence by a standardised test procedure (ICS) appears to be more authentic and reliable than a unique questioning. In our study, the ICS 24 h pad test shows no significant difference to the published data of other studies.

This represents a reliable and comparable status of continence in our patients after retropubic prostatectomy.

Loss of blood, body-mass index, age of the patient and state of disease are discussed as influencing state of continence. Sacco et al. could ascertain age, a non-nerve-sparing technique, and strictures of the anastomosis as risk factors for a post-prostatectomy-incontinence. This paper shows a longer follow-up (95 months) and a larger patient population (n = 1144), but is based on a comparable oncological cohort [21].

Here a multivariate analysis including blood loss, patient's age and disease-state could not identify a risk factor for post-prostatectomy incontinence (s. 4.1.2).

#### 4.2.2 Status of continence regarding age

The status of continence in our study population after radical retropubic prostatectomy did not differ significantly in patients younger and older than 70 years of age. Even the early and late status of continence did not show any significant differences. Nevertheless, it was noteworthy that patients suffering from PPI out of the older patient's group showed more urine loss in the ICS 24 h pad test.

Another group recently reported comparable results: This investigation found, that older patients reached though delayed a status of continence, but that age was no risk factor for a remaining incontinence. This could be determined in a multivariate analysis by Majoros *et al. *in 2007, which included 166 patients [26].

In comparative tests carried out in our data (Student`s t-test) between status of continence and age, no significant difference could be detected (p = 0.61).

#### 4.2.3 Status of continence and stadium of disease

No valid analysis of the status of continence could be performed with respect to the histologic stage of the disease because of the small study population. The shown trend indicated independence of the continence status and the histologic stadium of disease. This finding coincides with another recent published study. There the status of continence was compared to the expansion of prostate cancer (T2b to T3) after radical retropubic prostatectomy. The authors could not detect a significant difference (this study included 288 patients; [3]). Moreover, work from Ward and colleagues did not find a relation between the status of continence and the postal-surgical tumour stage (pT2 and pT3) [27].

### 4.3 Satisfaction of treatment

Patients felt well informed concerning about prostate cancer therapy (86.5%). Cosmetic results were satisfying in (88.1%). Comparable satisfaction values were found after radiotherapy. The only striking difference to the surgical approach is that fewer patients would undergo radiotherapy again. A possible explanation is that many patients believe themselves "not being operable any more"[27]. Satisfaction questionnaire showed a small number of patients undergoing therapy of erectile dysfunction (24%). Thus nerve-sparing prostatectomy should be performed whenever oncological possible.

## 5. Conclusion

Patients undergoing retropubic prostatectomy kept a stable QoL and stable body functions in general. Their emotional situation reached a high and stable level after the procedure. Complaints about typical symptoms of prostate cancer (especially urinary symptoms) stayed in a normal range and were independent of age. In our patients older than 70 years of age we found a mild reduction of QoL and a rising problem concerning dyspnoe. Therefore the indication of prostatectomy should be discussed critically concerning comorbidity.

It has to be admit that the study design and sample size is weak to draw general conclusions.

The results of the sexual symptom scores could not be used to draw general conclusions as well because of a match-pairs analysis, which resulted in a different amount of a nerve-sparing prostatectomy.

In conclusion retropubic prostatectomy represents an accepted and reliable procedure. Nevertheless the primary avoidance or therapy of erectile dysfunction should lie in the focus of surgeons.

## 6. Abbreviations

ASA: American Society of Anesthesiologists physical status score; EORTC: European Organisation for Research and Treatment of Cancer; GHS: Global Health Status; ICS: International Continence Society; PCa: prostate cancer; PPI: Post-prostatectomy-incontinence; PR25: Module of EORTC QLQ-PR25 specialized prostate cancer; PSA: Prostata-spezifisches Antigen; QLQ-C30: validated questionnaire for QoL; QoL: Quality of life; RPX: radical retropubic prostatectomy; Y: Year

## 7. Competing interests

The Authors declare that they have no competing interests.

## 8. Authors' contributions

TD performed interviews, pad tests and draft the manuscript. CM perfomed surgery and helped to draft the manuscript. AG helped in patients recruitment. MG performed surgery, designed the study and wrote the manuscript. All authors read and approved the final manuscript.

## Supplementary Material

Additional file 1 **Satisfaction questionnaire**. Patients attitude towards performed surgery was asked using a self-created questionnaire. Click here for file
