# Supplementary material for: Quality of life of patients after retropubic prostatectomy - Pre- and postoperative scores of the EORTC QLQ-C30 and QLQ-PR25
Source: Health Qual Life Outcomes. 2011 Nov 2;9:93. doi: 10.1186/1477-7525-9-93 (PMC3238231; doi:10.1186/1477-7525-9-93)
Supplement: Additional file 1 — Satisfaction questionnaire. Patients attitude towards performed surgery was asked using a self-created questionnaire. [file 1477-7525-9-93-S1.DOC]

Additional file 1: Satisfaction questionnaire

| 1. Would you choose the same therapy again? | Yes | No |
| --- | --- | --- |
| 2. Do you feel informed well about your disease? | Yes | No |
| 3. Has been a therapy of erectile dysfunction performed? | Yes | No |
| 4. Are you satisfied by the cosmetic outcome of surgery? | Yes | No |

Patients attitude towards performed surgery was asked using a self-created questionnaire shown above.
